# Using Gut Microbiota and Gut Metabolites as Biomarkers for Diseases in Monogastric Farm Animals

**DOI:** 10.3390/ani16142247

**Published:** 2026-07-21

**Authors:** Neil Ross McEwan, Gerardo Manuel Nava-Morales, Philippa Kate Morrison, Tercia Reis de Souza, Konisgmar Escobar-García, Samantha Elizabeth Bautista-Marín, Gerardo Mariscal-Ladín, Juan Carlos Silva-Jarquín, José Guadalupe Gomez-Soto, Andrea Margarita Olvera-Ramírez

**Affiliations:** 1 SRUC School of Veterinary Medicine and Biosciences, Scotland’s Rural College, Craibstone Estate, Aberdeen AB21 9YA, UK; neil.mcewan@sruc.ac.uk (N.R.M.); philippa.morrison@sruc.ac.uk (P.K.M.); 2 Facultad de Química, Universidad Autónoma de Querétaro, Las Campanas S/N, Querétaro C.P. 76010, Mexico; gerardomnava@gmail.com; 3 Cuerpo Académico Nutrición Salud y Producción Animal, Facultad de Ciencias Naturales, Universidad Autónoma de Querétaro, Avenida de las Ciencias S/N, Juriquilla, Delegación Santa Rosa Jáurgui, Queretaro C.P. 76230, Mexico; tercia@uaq.mx (T.R.d.S.); konisgmar.escobar@uaq.mx (K.E.-G.); samantha.bautista@uaq.mx (S.E.B.-M.); mariscal.gerardo@inifap.gob.mx (G.M.-L.); jcarlos.silva@uaq.mx (J.C.S.-J.); jose.gomez2@uaq.mx (J.G.G.-S.)

**Keywords:** biomarkers, gut microbiota, monogastric, farm animals

## Abstract

The use of biomarkers as diagnostic tools for animal health is becoming an increasingly important resource. In particular, this is true for digestive conditions, where an analysis of fecal samples can provide a non-invasive source of material for analysis. These samples can be used for the early detection of developing conditions or where an infection has taken place. However, biomarkers can also be used as a predictive tool for the advanced identification of animals that may be at an increased risk of developing a condition or having increased susceptibility to a particular type of infection. In the current narrative review, we focus on the use of both the gut microbiota and their resulting metabolites as biomarkers in monogastric farmed animals. Specifically, we review the existing knowledge available in the scientific literature for pigs, rabbits, horses and chickens.

## Simple Summary

A narrative review has been written to identify gut microbiota and their associated metabolites as biomarkers for different diseases in monogastric farm animals with the view to them being used as early diagnostic tools.

## 1. Introduction

Many aspects of healthcare, for both humans and domesticated animals, rely on being able to detect individuals that have a predisposition to a particular condition or are more likely to be susceptible to infection [1]. In doing so, it may be possible to take prophylactic approaches to reduce or eradicate these risks. Some approaches may involve genetic screening; others may involve an analysis of samples collected from animals [2]. These samples can be collected from a variety of different kinds of tissues, fluids or waste products, such as blood, saliva, urine, semen and feces.

In the current review, we concentrate on the use of fecal and digestive samples as a means of looking for biomarkers. Particularly, in the case of fecal samples, these have the advantage of meaning that the studies can make use of material that does not require collection using invasive procedures. Biomarkers in fecal samples can include both the types of microbes found in the stool sample and also some of the metabolites produced by either these microbes or the host animal [3].

In a medical context, fecal biomarkers have routinely become used as a mechanism for early detection of cancer in the lower gut [4,5,6], colitis [7] and inflammatory bowel disease [8,9]. In addition, fecal biomarkers can be used to study aspects of the microbiota–gut–brain axis [10], other digestive tissues [11] and even as a marker of food intake [12].

Previously, many of these digestive investigations have relied on obtaining digestive samples using invasive methods such as the use of cannulae being fitted at different points along the digestive tract of an animal (e.g., duodenal, cecal or ileal cannulae). However, some such experiments involving invasive procedures have received a certain level of public criticism for working with live animals, meaning that reduction, refinement and replacement approaches need to be considered as potential alternatives to the existing methods [13]. In the case of collecting intestinal samples, it has been proposed that, particularly in animals where fermentation takes place in the hindgut rather than the foregut, fecal samples may serve as alternatives to cecal or colonic samples. This includes work dealing with humans [14]. This premise relies on the assumption that although fecal samples will differ from samples collected from more proximal positions in the digestive tract, the potential loss of information in not using fecal samples will be more than offset by the use of more humane and non-invasive collection methods. This may be more likely to be a reasonable assumption for samples that would have been collected from the most distal parts of the digestive tract, but due to constantly ongoing metabolic processes at different points of the tract, fecal biomarkers may prove to be a poor reflection of the contents of more proximal regions such as the small intestine. More recent work suggests that there may be more of a difference between fecal samples and samples still within the digestive tract, as well as at different points within the tract, than was first considered. This is true for samples collected from horses [15].

Therefore, to achieve these objectives, it is essential to develop screening systems for fecal biomarkers that most reliably provide information on the intestinal regions of interest. In the current paper we provide a narrative review to explore what is known about the use of digestive biomarkers in monogastric (i.e., non-ruminant) farmed animals, specifically pigs, rabbits, horses and chickens.

## 2. Selection Criteria for Inclusion of Publications and Definitions

Web of Science, Google Scholar and ResearchGate were searched for candidate papers for inclusion in this review. Key words used were as follows:

(pig OR pigs OR scrofa OR porcine OR horse OR horses OR equine OR equid OR Equus OR rabbit OR rabbits OR cuniculus OR chicken OR chickens OR Gallus) AND

(digestion OR digestive OR caecum OR cecum OR cecal OR caecal OR intestine OR intestinal OR colon OR colonic OR faecal OR fecal OR feces OR faeces OR gastrointestinal) AND

(biomarker OR biomarkers OR metabolite OR metabolites OR metabolic OR microbiome OR microbes OR microbial OR microbiota).

Papers identified by these searches were checked for suitability for inclusion as reference material. Where a paper was used in the manuscript, the papers cited in the bibliography were also checked, partly as original sources of material cited within the papers, but also in case additional papers were identified that had not been found in the initial search process. In a similar manner, papers being used were checked for them having been used as a citation in a more recent publication, again as a precautionary measure in case papers were not being picked up in the initial trawl of the literature.

A fairly generic approach to the consideration of something as a biomarker was adopted. Biological markers used included the identification of genetic markers that can be linked to a condition or increased predisposition to a condition; the detection of a metabolite (either microbial- or host-derived); or the absence, presence or changes in abundance for specific microbes. While the emphasis was placed on using samples from feces or digesta, where appropriate, samples from biopsies, blood or urine were included in the narrative.

## 3. Gastrointestinal Functionality

Optimal gastrointestinal functionality should be regarded as an essential requirement for achieving sustainable and efficient animal production. This concept is based on the fact that the proper functioning of the gastrointestinal tract (GIT) is associated with maintaining the health of an animal, which in turn gives better animal production and keeps high levels of animal welfare. It is considered that several mechanisms are involved in the GIT’s functionality, and these interact in a dynamic and complex manner [16]. Conway [17] described that gut health depends on a combination of the diet, mucosa and microbiota. The GIT has a specialized role acting as the site of digestion and subsequent absorption from the diet. However, additionally, it plays a crucial role as a defense barrier against pathogens and toxins [18]. For these reasons, it has been proposed that gut health is best achieved when it is in “a stable state where the microbiome and intestinal tract exist in symbiotic balance and the animal’s wellbeing and its performance are not limited by intestinal dysfunction”. This definition integrates principal components such as the diet, the effective structure with the barrier function of the GIT, a stable microbiota, digestion and effective absorption of feed, a competent immune state, integrity of the intestinal mucosa and the neuroendocrine and motor functions of the intestine [16,19].

In terms of their digestive anatomy, pigs, rabbits, horses and poultry possess a system that is more simple than that seen in ruminants and camelids, in that they do not have the specialized foregut that allows animals such as cattle, sheep and llamas to engage in foregut fermentation. Instead, pigs, rabbits, horses and poultry have a single-chambered foregut, which primarily relies on enzymatic digestion to break down carbohydrates, proteins and fats [20,21]. However, there are still differences between these species in terms of anatomical organs distal to the glandular stomach (or proventriculus in the case of birds). For example, birds have a gizzard, which is a structure that is not present in mammals. Other differences include the nature of the cecal region, with birds having two ceca and pigs having a relatively simple cecum as befits an omnivore, whilst the horse and rabbit both have an enlarged cecum, which has evolved for the fermentation of plant material and the rabbit having an appendicular or vermiform cecum positioned at the tip of the main body of the cecum.

The gastrointestinal function also shows temporal variation. Possibly this is best illustrated by the transition required to move from a diet of milk in very young mammals through to the time when the animal is fully weaned. However, even this is an under-simplification of what happens, as the transition is gradual, moving from the early intake of colostrum in the milk in the first few days to a diet still primarily composed of milk to one where the animal is on a diet of both milk and solids and then ultimately an animal that is fully weaned.

The digestive tract of a mammalian fetus is sterile but from the birthing process onwards has the potential to become infected with microbes [22]. The types of microbes to which the newborn animal is likely to be exposed varies depending on the type of birth, being different between those born via a vaginal birth, versus those born via Caesarian-section [23]. Thus, the influence of the microbiota, and by implication the metabolites that they will produce, takes place from the first moment that a mammal leaves its mother’s body. During the early stages of life, mammals feed on milk, which is a highly digestible food and has bioactive factors with antimicrobial properties [24] that accelerate the development of the digestive system during the lactation period, preparing the animals for weaning [25]. For this reason, the intake of colostrum and maternal milk is essential for the development of the young animal. Simultaneously, in parallel with the development of the digestive system, there is a maturation of other vital systems such as the immune, circulatory, nervous and hormonal systems, and together they participate in the utilization of food by the animals [26,27,28], and there is cooperation between the microbiota of the digestive system and the host, which is essential to maintain good health and good production parameters [29]. Gradually the young animal moves to an inclusion of solids in the diet, which increases the secretory and absorptive capacity, especially in the mucosa throughout the digestive tract, and develops more rapidly due to the stimulation of solid foods, thus increasing the secretory and absorptive capacity [30]. The importance of ensuring that the young animals get access to solid food is reiterated by the fact that many breeders use creep feeding to ensure that the young animals can have exclusive access to solid food without allowing adult animals access to the food intended for young animals [31]. Having integrity of the small intestine mucosa and its cells, which are responsible for the absorption process, is key in digestive biology in later life [32]. Failures in the mucosa and accessory organs such as the pancreas and liver can compromise the digestive process in the small intestine, potentially resulting in a deficit in the supply of digestive secretions [33]. Thus, knowledge of metabolites such as these secretions and how they can be affected can be a key part of the understanding of digestive processes.

## 4. Microbiota

The microbiota constitutes a diverse ecological community in the gastrointestinal tract (GIT) of monogastric farm animals and varies across the different compartments of the gut due to environmental and physiological factors that favor the survival and proliferation of specific microbial populations. Therefore, the gut microbiota, which is the largest symbiotic ecosystem with the host, plays an important role in gut development, digestion, nutrition and immune responses [21,34,35,36]. The gut microbiota also has a role to play in the absorption and metabolism of feed, regulating gut motility and intestinal barrier homeostasis [37,38,39]. Microbial communities in the GIT can contain bacteria, archaea, fungi, protozoa and viruses, and the diversity depends on the host species, region of the digestive tract, age of host, sex of the host, diet and physiological or environmental factors [40,41]. As mentioned already, microbial colonization takes place shortly after birth, although its composition is not a fixed entity as it adapts and responds to changes over the course of the animal’s life, reaching a symbiotic relationship with the host animal as it consolidates its stability and functionality [40,42].

Of the species being discussed in this narrative, temporal variation in the microbiota of the pig has probably been studied in more detail than any of the others, probably due to pigs being used as models for medical research. In this example, the development of the gut microbiota in the piglets is gradual and sequential [43]. The earliest colonizers are *E. coli* and *Streptococcus* spp. Having them colonizing at an early stage gives a better anaerobic environment for other bacterial colonizers such as species within the genera *Bacteroides*, *Bifidobacterium*, *Clostridium* and *Lactobacillus* [44]. While these are appropriate for a suckling piglet relying on its mother’s milk, the introduction of a cereal-based diet at weaning causes a dramatic shift in the gut microbiota with an increase in the abundance of members of the genus *Prevotella* [45] and so the change in the pattern continues throughout the life of the animal [46].

Historically, species of microbes present in digesta or fecal samples were identified by growing them on agar plates. However, with recent advances in DNA sequencing technology it has become increasingly easy to study the composition of a microbial community without the need for growing organisms. Almost 20 years ago, the use of sequence information became increasingly important with the first large-scale DNA sequencing project, which resulted in around 62,000 sequences being identified from a human fecal sample [47]. In that particular example, the microbial community present was comprised primarily of Firmicutes (around 81% of the sequences), with the next most abundant being Actinobacteria (around 15%), and Bacteroidetes and Proteobacteria making up nearly all of the rest with about 2% each. The use of next-generation sequencing has since been applied to fecal samples from a number of different species, including the four animals considered in this narrative (chickens, horses, pigs and rabbits).

More recently, microbiota studies have been extended beyond the collection of fecal samples to other organs in the digestive tract (e.g., the cecum and ileum) [48,49,50,51,52]. Through these experiments, it was possible to demonstrate that cecal and ileal microbial populations are linked to intestinal health and animal performance, highlighting the necessity, where possible, to go beyond reliance on data from fecal samples for these types of studies. In addition to providing this information, the sequencing efforts underpinning it has resulted in the additional bonus of archiving millions of 16S rRNA gene fragment sequences, which are likely to be useful to microbiome analysis in the future.

**Figure 1:**
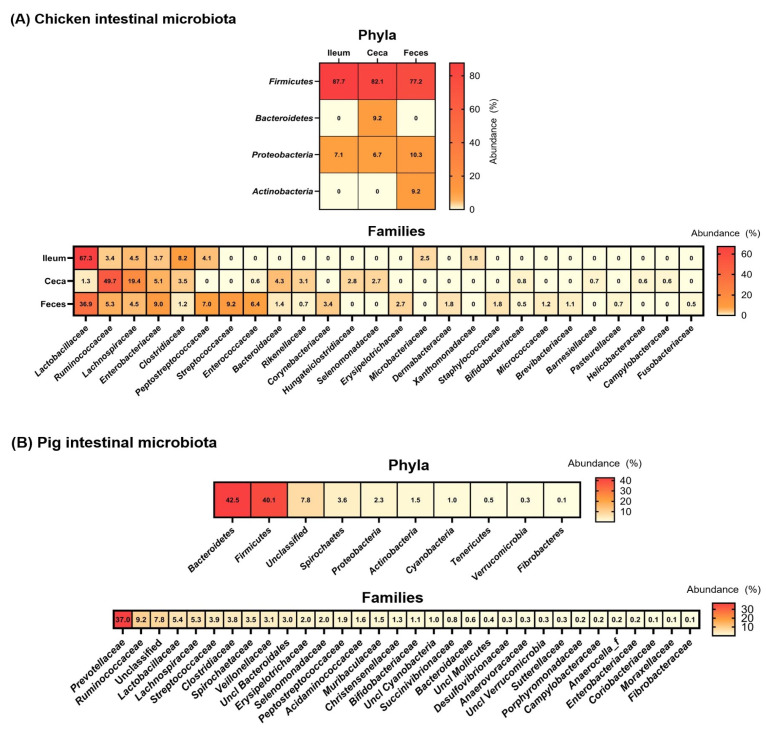
Diversity and abundance of core intestinal microbiota in chickens (**A**) and pigs (**B**). Heatmaps depicting the relative abundance in percentage (numbers inside the heat map and scale on the right side). This figure represents ~65 million and ~10 million 16S rRNA sequence reads obtained from [53,54].

Already these sequence datasets have allowed us to identify the core bacterial microbiota composition at different points along the digestive tract. For example, although the most abundant bacterial phyla present in the ileum, ceca and feces of chickens are Firmicutes, Proteobacteria, Bacteroidetes and Actinobacteria, the relative proportions of each changes with the different parts of the gut. In all three parts of the tract, the Firmicutes were the most abundant, ranging from 77% to 87% of the total sequences. Although most abundant in all three parts of the tract, the level of Firmicutes was most abundant in the ileum. The Bacteroidetes were at their most abundant in the ceca, and the Actinobacteria and Proteobacteria were at their most abundant in the feces [53]. Moreover, this dataset also revealed that 26 bacterial families represent the core intestinal microbiota in chickens, from which 8 families (*Lactobacillaceae*, *Ruminococcaceae*, *Lachnospiraceae*, *Enterobacteriaceae*, *Clostridiaceae*, *Peptostreptococcaceae*, *Streptococcaceae* and *Enterococcaceae*) represent at least 80% of the intestinal microbiota (Figure 1A).

In the case of the pig microbiome again, based on data from the Animal Microbiome Database [54], certain taxa of microbes dominated. However, unlike the chicken, no single phylum was considerably more abundant than all others, with both Bacteroidetes and Firmicutes each representing around 40% of the sequences, together with Spirochaetes, Proteobacteria and Actinobacteria being the next most abundant phyla. In addition, 32 bacterial families were found to represent the core intestinal microbiota in pigs, of which 9 families (*Prevotellaceae*, *Ruminococcaceae*, *Lactobacillaceae*, *Lachnospiraceae*, *Streptococcaceae*, *Clostridiaceae*, *Spirochaetaceae*, *Veillonellaceae* and *Erysipelotrichaceae*) collectively represented at least 80% of the intestinal microbiota (Figure 1B).

In the case of the horse, around 90% of the sequences obtained from the hindgut (cecum, large colon, small colon and feces) were from Firmicutes and Bacteroidetes, with a fairly even split at all sites (40% to 50%) for both phyla [55]. However, in the one site sampled, which lies proximal of the hindgut (the ileum), around 70% of the sequences were from Firmicutes and only around 10% were from Bacteroidetes. In addition, the ileum had around 15% of its sequences identified as being of Proteobacterial origin, whereas in the more distal areas, this value was considerably less, albeit they were present in all sample sites [55].

One of the earliest isolations of bacteria with cellulolytic activity from the digestive tract of the horse was published over 60 years ago [56]. A couple of decades later, an investigation of the bacterial community present in the cecum of the horse was performed using transmission electron microscopy to study the morphology of bacteria associated with plant tissues in the cecum of horses [57]. Numerous examples of both bacilli and cocci were observed as being in close proximity to sites on plants where erosion had taken place. Based on these observations, it was surmised that these microbes were also producing extracellular enzymes capable of breaking down plant cell walls.

It was almost a decade later before *Fibrobacter succinogenes* was identified in a pony by use of a molecular probe [58]. Shortly after that, *F. succinogenes* was again identified as being present in digestive samples from ponies and donkeys [59]. However, its abundance, as with *Ruminococcus albus*, was considerably lower than that seen for *Ruminococcus flavefaciens*, which has been described as the most abundant of the cellulolytic bacteria present in the equine gut microbial community. However, the true extent of the bacterial community in the digestive tract of the horse only became clear at the turn of the century when 272 random clones of 16S rRNA genes were sequenced [60]. This analysis resulted in the identification of 168 operational taxonomic units (OTUs), with 92% of the sequences being allocated to one of two phyla. The more abundant of these, low G+C Gram-positive bacteria accounted for 72% of all sequences and the Cytophaga–Flexibacter-Bacteroides phylum accounted for 20% of the sequences. In total, 37% of the sequences were found to belong to members of the clostridial XIVa cluster. While it had been expected that there might be some level of similarity between the bacterial population of the rumen and the equine hindgut, the reality was that only 5% of the sequences were from previously identified organisms, with a further 6% from unknown organisms whose sequences had been deposited in DNA databases. Thus, 89% of the sequences were from previously unrecorded sources. This pattern of differences between the rumen and equal cecal populations is one that has been repeated in other works, including those involving next-generation sequencing (e.g., [55]).

Unlike the other digestive systems investigated in this narrative, there are also eukaryotic microbes present in the equine digestive tract. From an early point in their studies, anaerobic fungi were identified as having strong cellulose-degrading capabilities [61] and colonizing the digestive tract at an early stage postpartum [62]. However, ciliated protozoa, which have been extensively described since the 19th century [63,64], were initially assumed to not have the ability to digest plant material [65], although more recently the isolation of cDNA molecules capable of degrading cellulose and hemicellulose from ciliates from the equine digestive tract suggests that at least some of them have this ability [66]. The significance of these ciliates is highlighted by the fact that their initial colonization of the digestive tract begins to take place within the first week of life following young foals engaging in coprophagic behavior towards their mother’s feces [67].

A similar pattern to that seen in the horse was seen in the microbial community of the rabbit. Early reports concentrated on work dealing with individual species that had been isolated from the digestive tract of the rabbit, particularly those that had previously been described in other gut ecosystems (e.g., [68,69]. However, once the first report of organisms based on 16S rRNA genes was published [70], it became clear that around half of the sequences identified were different from those already in the database, either in terms of that organisms that have already been described or for sequences previously reported but from unknown organisms. This suggested that there are bacterial species that appear to be specific to the rabbit, a point reiterated in a subsequent similar analysis [71] and more recently with the use of next-generation sequencing [72].

In the case of the rabbit’s digestive microbiome, the most abundant phylum present in fecal samples varied depending on their diet. The two most abundant were Firmicutes and Bacteroidetes. In the case of captive rabbits, these were present at approximately equal (around 30%) abundance in the fecal samples, whereas the samples from wild rabbits still had around 30% Bacteroidetes but around half were from Firmicutes. In both cases, the remaining sequences, other than low levels of Proteobacteria, were generally from unidentified organisms [72], which is a reflection of how poorly the rabbit microbiome has been studied relative to that of some of the other domesticated species. When the other areas of the digestive tract were studied in the wild rabbits, around half of the sequences were from Firmicutes and around 20% to 40% were sequences from Bacteroidetes. In all cases, the remaining sequences, other than low levels of Proteobacteria, were generally from unidentified organisms, with the exception of low levels of Tenericutes being observed specifically in the samples from the jejunum [72].

While there is increasing literature on the bacterial community of the digestive tract in the rabbit, there is no such equivalent literature on eukaryotic microbes from this environment. Although this is another environment involved in the hindgut fermentation of a herbivorous diet, the reason for their absence is that although there are papers dealing with protozoa in the digestive tract (e.g., [73,74], as well as fungi (e.g., [75]), neither fungi nor protozoa are regarded as being members of the core microbial population of the rabbit, but rather they are considered transient organisms.

Thus, studies in all four organisms investigated in this narrative show that there are differences in the bacterial composition of different parts of their tract.

## 5. Intestinal Immune System

As already mentioned, the small intestine is where some of the most important parts of digestion take place, but it is also an important immune organ, consisting of a complex cellular network, secreted peptides and proteins and other host defenses [76,77]. The intestine mucosal immune system has three different mucosal lymphoid structures, Peyer’s patches, the lamina propria and the epithelia [78,79], and is composed of innate and adaptive constituents.

Innate immunity plays a central role in intestinal immune defense against invading pathogens and serves as a bridge to the activation of the adaptive immune system [77]. The role played by the innate immunity has a knock-on effect in terms of it being able to influence the microbiota present within an individual and by inference with the range of metabolites that can be produced by this microbial population. The innate immune system includes cells such as macrophages and dendritic cells, which ultimately have a role to play in controlling pathological inflammation [80]. It also contains eosinophils, which play an important role in terms of promoting tissue development, cellular communication and expression of a variety of receptors and bioactive molecules [81]. Paneth cells are also part of the innate immune system and produce antimicrobial peptides that are able to help to regulate which types of bacteria are present within the tract [82]. Hence, the cells of the innate immune system help to prevent inflammation, promote cellular communication and produce molecules that control the content of the microbial community and thereby control the metabolites and biomarkers they produce.

The adaptive immune system has components that interact with the innate immune system and help to confer long-lasting immunity and to mount an effective immune response [83]. Within the adaptive immune system are the gut-associated lymphoid tissues (GALTs), which contain the immune cells that undergo initial priming and differentiation. In most mammalian species the GALT is adjacent to the epithelium throughout the gut and is most abundant in the Peyer’s patches. However, in rabbits, the GALT is even more developed than in other mammalian species, since they possess two organized lymphoid-tissue-differentiated segments or organs, the sacculus rotundus and the vermiform appendix (or appendicular cecum), which account for 50% of the total lymphoid tissue in the rabbit [84]. The GALT is also well-developed in chickens as it is composed of Peyer’s Patches, Meckel’s diverticulum, two cecal tonsils, diffuse lymphoid tissue in the rectum, the bursa of Fabricius and diffuse lymphoid tissue in the wall of the proctodeum [85].

Thus, the effects of the immune system are able to impact not only the cells of the digestive tract, but also the microbes found within the digestive tract. In turn, this impacts the metabolites and biomarkers that can be produced by these cells.

## 6. Fecal Biomarkers

As mentioned above, there are variations between the digestive tracts of the different species discussed in this narrative. As a consequence of this, there are different microbes present in the different tracts, and as a result of this, different metabolites and biomarkers are produced. In this section, the biomarkers will be discussed species by species, with cross-referencing between species where appropriate.

The use of diagnostic approaches, based on biomarkers, is now sufficiently refined to allow the method to detect the presence and abundance of potential pathogens, even when there are no clinical symptoms. In pigs, this has been shown to be the case for both bacteria such as *Clostridium perfringens* type A and *Lawsonia intracellularis*, as well as for viruses such as Rotaviruses [86]. This high level of sensitivity may initially appear to be advantageous, particularly if it can be used to detect individuals at risk, prior to them developing clinical symptoms. However, this needs to be treated with caution and on a case-by-case basis as some microbes are part of the natural community but can cause problems when they reach an elevated level. For example, *Clostridium* spp., *E. coli* and *Salmonella* spp. are all known to be capable of causing digestive disorders but equally can be part of the normal digestive microbiome of the pig; albeit, this will often be at subclinical levels [87]. The issue can be compounded further by a clinical symptom potentially arising from a number of potential sources, and it can be difficult to pinpoint which was the causal agent in a particular case. For example, where piglets from a single farm were investigated for a range of potential pathogens, relative to their diarrhea status [88], i.e., those that were suffering from diarrhea versus those which were clinically healthy, in all outbreaks, more than one potential diarrhea-causing pathogen could be detected using PCR-based techniques applied to fecal samples. However, of the potential causal agents, only Rotavirus A was statistically correlated with animals with diarrhea, while Rotavirus B, Rotavirus C, porcine epidemic diarrhea virus, transmissible gastroenteritis virus, *E. coli*, *Clostridium perfringens* and *Clostridioides difficile* (previously known as *Clostridium difficile*), while detected in some animals, showed no statistical correlation with animals suffering from diarrhea. Thus, biomarkers can be used as a means of identifying individual species, which can help with the detection of conditions.

Moreover, it has been recognized that certain pathogens, although detectable in diarrhea, are more likely to normally occupy specific regions of the digestive tract [86]. For example, *Salmonella* Typhimurium is most likely to be found in the ileum, cecum or colon, whereas *C. perfringens* and Rotaviruses are more likely to be found in either the jejunum or ileum. Thus, the use of biomarkers requires determining the type of marker to look for, the levels it can be detected at and also the regions of the digestive tract to check for each possible pathogen. In the case of pathogens most typically found in the more proximal areas of the digestive tract, this may minimize or even preclude their potential use as a fecal biomarker.

### 6.1. Pigs

Pigs will be considered first as they are probably best studied. In part, this is because they are often used as a model for medical research due to them having some similarities to humans, a relatively large mammalian species with an omnivorous diet. Moreover, they have similar metabolic and intestinal physiological functions [89]. Therefore, investigation of digestive biomarkers in pigs has the potential to contribute beyond just pig nutrition and healthcare, with the possibility of providing information that may have medical implications.

#### 6.1.1. Pig Genome Markers

Even from an early age in the piglet, biomarkers can be used to monitor predisposition to necrotizing enterocolitis [90]. Three genes in particular have been identified as meriting further investigation: AOAH (acyloxyacyl hydrolase), FKBP5 (which acts on the glucocorticoid receptor complex) and PAK2 (which encodes a serine/threonine kinase). In particular, the use of PAK2 may seem surprising since it is associated with multiple cellular processes, including cell motility, cell cycle regulation and cell proliferation, and defects in the gene have been described as being associated with ocular problems [91] and also with myelination [92]. FKBP5 has been shown to have effects relating to intervention for stress-related disorders [93]. However, it is known to have pleiotropic effects, and it also acts on glucocorticoid receptors, which play a role in a number of key cellular processes. AOAH has been shown to transform lipopolysaccharides (LPS) or endotoxins and, in doing so, reduces tissue injury and death from infection [94]. The effect of this prevents LPS produced by microbes of the gut from entering the bloodstream.

#### 6.1.2. Pig Gut Microbes as Biomarkers

The microbial community present in the digestive tract is a reflection of the organisms best able to make use of the nutrients available to the host animal at any particular time. In turn, these microbes will produce certain products and so will influence the composition of the digestive metabolic profile. However, since the diet of an animal can, and does, change over time, the use of microbes and the microbial metabolites can only serve as a temporal feature, and the biomarkers will change with time.

However, it is important to remember that the use of biomarkers is a temporal feature and may change over time. A good example of the changing nature of biomarkers was observed in the microbiome of weaning piglets [95] at three different ages (14, 21 and 28 days). These dates coincided with critical time points in the life of the animal, namely at a time that was pre-weaning, the day of weaning and a post-weaning date, respectively. Their investigation included an analysis of bacterial population shifts, changes in the bacteriophage population and blood serum metabolites. In all animals at each of the three ages, the main bacterial genera in fecal samples were *Bacteroides* and *Prevotella*, both of which are members of the Order Bacteroidales, with *B. fragilis* as the most abundant species in all cases. Although *B. fragilis* is the major species in feces, the most abundant genus is actually *Prevotella* which is present at 41% and 35% of the total abundance for the proximal and distal colon, respectively, in adult pigs [96].

The results seen in the piglets suggest that, based on their fecal microbial population, the approximate abundance of many genera of bacteria is laid down early in life. However, it is becoming more clear that fecal samples cannot realistically give an indication of the bacterial population in the more proximal areas of the piglet’s digestive tract. For example, in the small intestine, the most abundant organisms in adult pigs are Gram-positive organisms, such as members of the genera *Lactobacillus* and *Clostridium* [96]. In this particular example, the organisms that could be regarded as biomarkers for piglets at different stages in their lives were as follows: *Comamonas kerstersii*, *Comamonas aquatica* and *Roseburia inulinivorans*, which were enriched at day 14, with *Clostridium perfringens* and *Ruminococcus flavefaciens* enriched at day 21. However, *P. copri* was the only significantly enriched species in the day-28 group. Thus, this demonstrates that it is not just necessary to identify bacteria that can be used as biomarkers but also determine their role in a temporal manner.

Digestive capacity increases with age [26]. However, at weaning, the capacity to digest plant-based ingredients is still small, and the abrupt change from milk to solid foods during weaning, in addition to all the other stressors of weaning, can lead to an imbalance of the microbiota [29] and severe digestive problems, weight loss and even death. Therefore, the targeted control of dietary components to promote the establishment of a healthy bacterial community is an important method to prevent nutritional diarrhea in weaned piglets [97]. In this particular example, manipulation of the levels of dietary fiber can lead to alterations of the production of short-chain fatty acids (SCFA) and other metabolites, including a decrease in toxic metabolites in response to the change arising from the changes to the microbial population.

Porcine diarrhea causes several economic problems in the pig industry because it leads to a decrease in average weight gain, increases the use of medication and causes mortality, principally in piglets [98,99,100]. Several authors cited by Knecht et al. [29] state that one of the main stress factors in piglets, which affects the destabilization of the intestinal microbiota, is weaning, which causes a decrease in the diversity and number of microbial populations in the gastrointestinal tract. Furthermore, during the weaning period, the intestinal microbial composition and immune system are still developing, making them susceptible to pathogens that cause post-weaning diarrhea [42].

Neonatal piglet diarrhea has been associated with increases in the relative abundance of *Prevotella* (*Bacteroidetes*), *Sutterella* and *Campylobacter* (*Proteobacteria*) [101] and *Enterococcus* (*Firmicutes*) and is more abundant in neonatal porcine diarrhea-affected piglets [102]. Sun et al. [98] collected swabs from piglet samples during lactation, weaning and post-weaning, and they identified fecal microbiota via 16S rRNA gene V4 region sequencing using an Illumina MiSeq platform. They found that the family *Enterobacteriaceae* was a biomarker in piglets during lactation, but the *Bacteroidales* family *S24–7* group was a biomarker in later stages of growth. In addition, *Escherichia-Shigella* was the core group in the diarrheic gut microbiota, whereas *Provteollaceace UCG-003* was the core group in the fecal microbiota of non-diarrheic piglets.

Post-weaning diarrhea represents a worldwide challenge for the pig industry and historically antibiotics. Different levels of dietary crude protein can help to reduce the incidence [103]. This changes the pattern of protein fermentation in the hindgut, which in turn influences the level of many metabolites being produced. These include SCFA, as well as sulfur-containing metabolites and aromatic compounds, meaning that these metabolites can be used as biomarkers. In addition, high doses of zinc oxide and copper sulfate have been used to control the diarrhea [103]. However, the development of bacterial antibiotic resistance and environmental contamination have generated interest in alternative strategies that can replace the use of undesirable practices.

At weaning, switching piglets from milk to solid feed is frequently associated with severe growth depression and diarrhea, leading to the so-called “post-weaning diarrhea syndrome,” which has a multifactorial etiology (management, diet composition and hygiene, etc.). Anorexia and undernutrition are the most important triggering factors of this syndrome [103,104]. The main causes of diarrhea are highlighted by high levels of crude protein, stimulation by certain antigenic proteins, high acid-binding capacity and contamination with mycotoxin deoxynivalenol in the diet.

Most studies conducted during the weaning transition report a decrease in *Lactobacillus* bacteria and a loss of microbial diversity, while *Clostridium* spp., *Prevotella* spp. and facultative anaerobes such as Proteobacteriaceae, including *Escherichia coli*, were positively affected [42]. This dysbiosis observed at weaning is now recognized as part of the etiology of post-weaning diarrhea.

Mechanistically, diarrhea in piglets is due to an imbalance in the absorption and secretion of intestinal fluids and electrolytes. This is generally due to enterotoxigenic *E. coli* (ETEC) and other bacteria such as *Clostridium perfringens* types A and C, *Clostridioides difficile* (previously known as *Clostridium difficile*), *Enterococcus* spp., *Salmonella* spp. or diarrheal viruses such as Rotavirus and Coronavirus [86]. These organisms can cause diarrhea when fluid secretions exceed the absorptive capacity [103]. López-Colom et al. [105] mention that the stress of weaning in piglets raises the opportunity for digestive pathogens to invade and colonize the immature developing gut. To explore this further, they evaluated the usefulness of three inflammatory and gut-wall-integrity serum biomarkers: intestinal type fatty acid binding protein (I-FABP), tumor necrosis factor alpha (TNF-α) and a main acute phase protein in pigs (PigMAP). These were investigated to assess the degree of intestinal histo-morphological damage in piglets that had been orally challenged with *Salmonella* Typhimurium or enterotoxigenic *Escherichia coli* K88. They found that I-FABP is useful as a biomarker of gut barrier integrity anticipating advanced tissue injury or illness development, together with either Pig-MAP or TNF-α. However, I-FABP and TNF-α are not exclusive for pig intestinal inflammation; but rather, the combination of all three biomarkers can be a non-invasive tool suitable for determining the degree of intestinal injury in weanlings.

Swine dysentery includes severe mucohemorragic diarrhea, caused by sphirichaete beta-hemolytic strains of *Brachyspira hyodysenteriae*, *Brachyspira hampsonii* and *Brachyspira suanatin.* They initially invade the goblet cells, causing excessive mucous secretion, or alternatively, the invading bacteria are preceded by initial damage to tight junctions. Clinical signs range from mild, mucous diarrhea and an unaltered general condition to severe hemorrhagic diarrhea with a mortality rate of 50–90% [106,107,108,109]. The dysentery is expressed when there are more specific anaerobic microbiota, such as *Fusobacterium necrophorum*, and when dietary modifications alter the colonic microbiota [110,111]. Depending on the types of microbes present, this can lead to a change in the metabolites being produced. For example, an increase in the quantity of protein reaching the colon can change some of the metabolites being produced, including metabolites that can be toxic. These include increases in the levels of ammonia and volatile phenols, which can be used as metabolic biomarkers [110].

Colitis is inflammation in the colon, which is a consequence of a complex biological defense mechanism against harmful stimuli such as infection by pathogenic bacteria and physical damage [112,113]. It can, at least in part, be kept in check by reducing dietary crude protein to 19% and in so doing reducing the level of toxic metabolites such as high ammonia levels [112]. Wilberts et al. [114] used a randomized complete block experiment to examine the effect of increased dietary fiber, through the feeding of distiller’s dried grains with solubles (DDGS), on the incidence of *Brachyspira*-associated colitis in pigs. They found that pigs receiving 30% DDGS shed, on average, one day prior to and developed dysentery nearly twice as fast as pigs that received no DDGS. Also, the study reported that the average pH of cecal and colonic content samples from pigs fed 30% DDGS for 5 weeks was significantly more alkaline relative to pigs fed no DDGS. They suggested a reduction in insoluble fiber through reducing or eliminating DDGS in swine rations. This should be considered an integral part of any effective disease-elimination strategy for swine dysentery. In addition, Burroug et al. [110] collected colonic contents at necropsy from pigs fed either 30% or no DDGS, which were analyzed to determine the relative abundance of bacterial taxa associated with feeding this ingredient. No difference in alpha diversity (richness) was detected between diet groups. However, the beta diversity was significantly different between groups with feeding of DDGS being associated with a decreased Firmicutes:Bacteriodetes ratio and a significantly lower abundance of *Lactobacillus* spp. Predictive functional profiling of the microbiota revealed more predicted genes associated with carbohydrate metabolism, protein digestion and the degradation of glycans in the microbiota of pigs fed DDGS. Taken together, these findings confirm that alterations in dietary insoluble fiber significantly alter the colonic microbial profile of pigs and suggests that the resultant microbiome may be predisposed to the development of colitis.

Burroug et al. [115] analyzed pigs with and without dysentery via metagenomic analysis using the V4 region of the bacterial 16S rRNA gene, and they found that the relative abundance of *Brachyspirales*, *Campylobacterales*, *Desulfovibrionales* and *Enterobacteriales* was higher in pigs with dysentery for both mucosal scrapings and luminal samples, while *Clostridiales*, *Erysipelotrichales* and *Fusobacteriales* were significantly more abundant in the luminal contents. In contrast, pigs without dysentery showed a higher abundance of *Burkholderiales* in both sample types, *Bacteroidales* and *Synergistales* were more abundant in scrapings and *Lactobacillales* and *Bifidobacteriales* were more abundant in luminal contents. At the genus level, the relative abundance of *Brachyspira*, *Campylobacter*, *Fusobacterium* and *Mogibacterium* was higher in pigs with dysentery for both mucosal scrapings and luminal samples, while *Desulfovibrio* was more abundant in scrapings and *Flexispira* was more abundant in the luminal contents.

Therefore, these studies identify fecal biomarkers of dysentery including *Desulfovibrio*, *Campylobacter*, *Mogibacterium* and *Fusobacterium* and biomarkers of the absence of dysentery development mediated by other organisms, e.g., *Lactobacillus*, *Bifidobacterium* and *Roseburia*.

### 6.2. Poultry

In poultry production, the GIT is a central regulator of digestion, nutrient absorption and immune defense, but it is also highly susceptible to dietary and environmental challenges. The gut microbiota and its metabolites are increasingly recognized as valuable biomarkers for health, productivity and resilience against disease. These biomarkers include the composition of the microbial community, SCFA profiles, mucin expression, cytokine levels, oxidative stress indicators and mineral metabolism parameters [116,117]. As was seen with the pigs, the effect of the diet in poultry can have an impact on the digestive microbial community and in turn the metabolites they produce. For example, the inclusion of phytochemicals, in particular polyphenols, can influence the polyphenol content and also the metabolites produced from the polyphenols, which in turn can impact the microbial community in chickens, in addition to leading to health-promoting benefits [116]. In the healthy gut, resistant starch and soluble dietary fibers are fermented by microbes in the ceca, leading to the production of SCFAs (primarily acetate, butyrate and propionate [117].

In broilers, the gut microbiota is affected by several factors, such as age, diet/feed type, breed, gender, hygiene, housing conditions, litter type and maternal factors, as well as probiotics, prebiotics, phytobiotics and phages [118]. Typically, these are dominated by members of the genera *Lactobacillus*, *Bifidobacterium* and *Faecalibacterium*, which are associated with the epithelial integrity, SCFA production and immunomodulation [119,120]. Dysbiosis in broilers is an intestinal imbalance, which compromises gut health, leading to impaired growth and increased disease susceptibility and welfare problems [121,122]. It is characterized by an overgrowth of *Clostridium perfringens* or *Escherichia coli* and has been associated with necrotic enteritis and intestinal inflammation [91,123,124,125]. The onset of necrotic enteritis is associated with a shift in the microbiota present within the GIT and is more a consequence of the *C. perfringens* multiplication and necrosis. Pathogenic *C. perfringens* strains interact and compete with other microbiota in the gut, and this interaction can affect disease induction and severity [126]. The effects can be multifactorial, but in terms of the current review, it is a drop in the abundance of members of the Ruminococcaceae family, which are responsible for much of the production of the SCFA butyrate [126]. This metabolite is considered to be anti-inflammatory, and its presence helps to stabilize the integrity of the intestines [126]. Yan et al. [127] reported a large group of ileal microbes that are significantly correlated with necrotic enteritis severity: Firmicutes, *Lactobacillus reuteri*, *Subdoligranulum variabile*, *Mediterraneibacter* spp. and *Staphylococcus* spp., as well as two genera of Actinobacteria (*Corynebacterium* and *Kocuria*) and two highly related Cyanobacteria. Also, Firmicutes, such as *Weissella*, *Romboutsia*, *Kurthia*, *Cuneatibacter*, *Blautia* and *Aerococcus*, appeared to be much more sensitive and rapidly declined in chickens even with mild necrotic enteritis. On the other hand, *Enterococcus cecorum* and two *Escherichia/Shigella* species were only enriched in the ileal microbiota of chickens with extremely severe necrotic enteritis, while several other species such as *Streptococcus gallolyticus* and *Bacteroides fragilis* remained unaltered by necrotic enteritis. In addition, dysbiosis in the mycobiota (the fungal population) has been described as decreasing the microbiota in necrotic enteritis in broilers, and Yang et al. [128] reported that the total bacterial population in the ileum increased by 2- to 3-fold in necrotic-enteritis-affected chickens, and the total fungal population progressively declined in more exacerbated necrotic enteritis [128]. Monitoring shifts in the microbial community composition therefore provides useful biomarkers of intestinal health. However, many non-invasive biomarkers are under investigation for broiler gut health assessment, but challenges remain in validating these markers due to the complexity of the gut ecosystem [129].

On the other hand, in conventional fast growing broiler flocks, performance parameters such as the feed conversion ratio and daily growth rate are often negatively affected by a dysfunctional digestive system such as dysbiosis and heat stress, which can cause intestinal abnormalities [130,131]. In the chicken, a negative correlation between performance parameters and Enterobacteriaceae expansion has been reported. Hence, the quantification of Enterobacteriaceae can be used to identify dysbiosis in poultry [132,133]. Also, ovotransferrin has been suggested as a candidate protein serum and fecal biomarker for intestinal health evaluation [134,135]. Ovotransferrin is a glycoprotein with an important role in the innate immune system and has antimicrobial, immunomodulating and anti-oxidant activities [136]. Rysman et al. [137] investigated ovotransferrin as a fecal biomarker under field conditions, by evaluating its association with broiler performances (individual body weight, FCR, average slaughter weight, mortality percentage, daily weight gain [g/d], the European production index and gut histological parameters such as the villus length, crypt depth and CD3^+^ T-cell infiltration in the gut wall). They found ovotransferrin in feces making it appear to be a metabolite that can act as a valuable indicator of the key phenomena underlying intestinal health issues in broilers. And they considered that ovotransferrin quantification might be a valuable tool to predict performance.

Broiler ascites syndrome is a common nutritional and metabolic disease in rapidly growing commercial broilers. Many factors are associated with the occurrence of ascites syndrome, such as the diet, management, environment and genetics. It is also known that low-temperature, high-energy, high-protein and high-salt diets are the main feeding and management factors leading to ascites syndrome [138,139]. Hong et al. [140] demonstrated that ascites syndrome increases the abundance of Firmicutes, Proteobacteria, Actinobacteria, Firmicutes-*Clostridium* and Gamma proteobacteria in the intestinal tract of rats while concurrently reducing the abundance of Bacteroidetes, Spirochaetes, Bacteroidia, Spirochaetia and bacilli. More recently, Rheman et al. [141] demonstrated, in an LEfSe analysis, that ascites syndrome reduced the abundance of Actinobacteria and increased the abundance of Bacteroidetes at the phylum level. In addition, they mention that metabolic diseases can impact the gut microbiome due to them having an impact on the production of beneficial metabolites such as SCFAs and vitamins. On the other hand, the antioxidant properties of prebiotics are also relevant. Fructans can reduce oxidative stress by promoting antioxidant metabolite production and upregulating endogenous defenses such as glutathione peroxidase and superoxide dismutase [142]. Biomarkers of this effect include decreased lipid peroxidation (TBARS, Thiobarbituric Acid Reactive Substances) and an improved hepatic redox balance [143]. These effects are particularly beneficial in broilers, where oxidative stress contributes to ascites syndrome. Birds predisposed to ascites display elevated interleukin-6, oxidative damage and mitochondrial dysfunction [144]. Dietary supplementation with fructans has been reported to mitigate these alterations, reducing inflammation, enhancing antioxidant activity and improving oxygen utilization, thereby decreasing ascites incidence [140].

SCFA profiles, especially acetate, propionate and butyrate, are important metabolic biomarkers of microbial metabolism. Their levels reflect fermentative activity, the intestinal energy supply and mucosal health [145]. Butyrate enhances epithelial cell turnover, stimulates goblet cells to secrete mucins and reinforces tight junctions, thus protecting the intestinal barrier [145,146,147]. Prebiotics such as inulin (agave-derived fructans), FOS and GOS modulate bacterial groups such as *Lactobacillus* and *Bifidobacterium* species, increase SCFA production [142] and improve the villus height and crypt depth ratio, along with goblet cell density [148,149], all of which can be considered histological biomarkers of improved gut function. At the immunological level, fructans increase IgA secretion, elevate anti-inflammatory cytokines (IL-10) and downregulate pro-inflammatory mediators (IL-6, TNF-α), supporting mucosal and systemic immunity [150,151,152,153]. In laying hens, prebiotics provide benefits that can be monitored through specific biomarkers. Fructans improve the microbial balance, increase cecal SCFA concentrations and stimulate mucin secretion, which support intestinal integrity throughout the laying cycle [154]. Systemically, hens supplemented with fructans show reduced oxidative stress biomarkers, which is relevant since a chronic oxidative imbalance contributes to lower laying persistency, poorer eggshell quality and reproductive aging [155,156]. A particularly important benefit in laying hens is the effect of prebiotics on calcium metabolism, which is essential for eggshell formation and skeletal health. Fructans enhance calcium absorption by reducing luminal pH through SCFA production, thereby increasing the solubility and bioavailability of minerals [157]. They also stimulate paracellular calcium transport and may upregulate calcium-binding proteins, contributing to improved mineral utilization. Biomarkers of this effect include higher plasma calcium, increased tibia ash content, improved bone mineral density and greater eggshell thickness and strength [16,158]. These improvements help prevent osteoporosis and fractures in older hens, while supporting egg quality and productive longevity.

In summary, poultry health can be effectively monitored through multiple biomarkers, the microbial community composition, SCFA profiles, mucin secretion, oxidative stress indicators, cytokine expression, immunoglobulin levels and calcium-metabolism parameters. Also, prebiotics such as fructans represent a sustainable nutritional strategy that improves gut integrity, reduces oxidative stress, enhances immunity and lowers the risk of ascitic syndrome in broilers. In laying hens, fructans further improve calcium utilization, eggshell quality and skeletal health, thereby supporting both productivity and animal welfare.

### 6.3. Rabbits

In many countries, rabbits are regarded as pets, but in other countries, the rabbit is farmed for its meat. Particularly, in some countries in Eastern Asia, South and Central America and Mediterranean nations, rabbit meat can constitute up to around 10% of meat consumption, therefore justifying their inclusion as a farmed animal.

One of the earliest examples of recognition of a digestive biomarker in rabbits dates back over 20 years when it was observed that *Clostridium perfringens* was often found in the fecal samples collected from animals with Epizootic Rabbit Enteropathy (ERE), a condition that includes symptoms such as diarrhea. Around this time, it was observed as having proliferated in animals in the field [159], and it was also observed that around 80% of ERE-affected animals in Belgium and the Netherlands had *C. perfringens* [160]. The potential use of *C. perfringens* as a biomarker for ERE was further developed based on the observation that there was a significant correlation between the alpha toxin produced by *C. perfringens* and lesions resulting from ERE [161]. Although there was a clear link between ERE and *C. perfringens*, attempts to induce ERE via inoculation with *C. perfringens* were unsuccessful [160,162,163], suggesting that although there is some form of association between the two, *C. perfringens* is not the primary cause of ERE.

ERE is assumed to be caused by a bacterial source, based on the frequent success of treatment with antibiotics. In addition, differences in microbiome population profiles could be seen between infected and non-infected animals using bacterial primers for PCR [164]. However, the causal agent, or agents, still remains unclear. Irrespective of that, the changes to the microbiota can still be used as potential biomarkers to indicate a possible ERE infection. These include a decrease in the members of the class Clostridia, with an associated increase in members of the class Bacteroidia [165]. In addition, the rabbits showing signs of ERE had elevated levels of Proteobacteria (mainly γ-Proteobacteria) and an increased number of Verrucomicrobiae, particularly members of the genus *Akkermansia*, primarily *A. muciniphila*. However, although these observations can be regarded as biomarkers for animals with ERE, they have not actually been shown to be the bacteria responsible for the development of ERE.

Relative to other domesticated species, the microbiome of the rabbit is still poorly characterized. In part, this is due to fact that many of the organisms detected in the first attempts to characterize the bacterial population via DNA sequencing identified several sequences from previously unidentified species [60,71]. Since they have only been identified based on the sequence of their 16S rRNA genes and have not been grown in cultures, this means that many have unknown metabolic properties. However, since the first identification of these organisms, metabolic functions have been assigned to some of them, including those closely related to species found in the digestive tracts of either ruminants or horses. In turn, the identification of some of the organisms associated with beneficial properties has led to an assessment of the roles played by dietary probiotics. For example, supplementing with *Bacillus cereus* var *toyoii* decreased the abundance of *Clostridium* spp. [166] and improved the general health of the animals [166,167]. The inclusion of live *Saccharomyces cerevisiae* increased the relative abundance of *Ruminococcus albus* [168], an organism known to play a role in breaking down cellulose, and was also found to generally improve the health of the rabbit [169]. While it may be argued that the identification of changes in the abundance of these organisms may not constitute biomarker identification in the strictest sense, detecting changes in the abundance of these organisms may serve as an indicator of the potential success of certain dietary supplements performing a probiotic role.

Where rabbits have been kept in low-hygiene conditions, this can lead to a decrease in members of the family Lachnospiraceae in the cecum [170]. This is the second most abundant bacterial family in the cecum (with Ruminococcacea, another member of the Firmicutes phylum, being the most abundant), and in turn, this affects the Ruminococcaceae/Lachnospiraceae ratio, with a significant increase in this value indicating that the animal has been kept in poor-hygiene conditions. When studied at the genus level, this was generally also accompanied by a specific increase in the abundance of bacteria from the Ruminococcaceae UCG-008 group. However, these observations were made from cecal samples, with nothing to indicate if these observations were also detectable at the fecal level.

An investigation using similar markers was performed around the same time [171] where caecotrophs were collected and analyzed. While caecotrophs (soft feces) and hard feces have different properties, working with caecotrophs allows sample collections without needing invasive methods. In this work, the unhealthy animals were ones suffering from ERE, and again bacteria were used as biomarkers. One of the most interesting observations in the caecotrophs from animals was the increase in members of the genus *Bacteroides*, which rose from around 7% to almost a third of the species present. This was also accompanied by a trend that was the opposite of that seen with the cecal samples; members of the Ruminococcaceae family (particularly members of the *Ruminococcus* genus) decreased, while members of the Lachnospiraceae family (particularly members of the *Blautia* and *Dorea* genera) increased. It is unclear how much of this is due to differences between cecal contents versus caecotrophs and how much is due to differences between ERE versus general poor hygiene. Nevertheless, it may serve to illustrate that it is important to use different biomarkers for different analyses.

A number of other investigations into the composition of microbiomes as a way to compare ERE versus non-ERE animals have highlighted a range of potential biomarkers, although, in many situations, this involves using cecal rather than fecal samples. For example, both *C. perfringens* and non-enteropathogenic *Escherichia coli* were found in fecal samples collected from animals infected with ERE [161]. Members of the genus *Clostridium* were also found to be associated with ERE in more recent work [172], together with the species *Cloacibacillus porcorum* and *Akkermansia muciniphila*, indicating their potential use as biomarkers for ERE. *Cloacibacillus porcorum* is an organism that was initially isolated as a species from pigs and was shown to be mucin-degrading and able to ferment amino acids [173] whilst *Akkermansia muciniphila*, which is also a mucin-degrading organism, was first identified in human fecal samples [174].

The analysis of the potential biomarkers can be clouded further though. For example, where rabbits that had recovered from ERE were compared with animals that had never suffered from ERE, although the animals appeared to be physically the same, there were differences in the microbiomes for both fecal and caecotrophic samples, which appear to have been as a result of some rabbits having previously been affected by ERE [175]. Similar differences were also observed for metabolites, most notably all of the short-chained fatty acids, both as a total and individually.

In the wider context of digestive diseases in general, a number of different bacterial species have been identified as putative biomarkers for affected rabbits [176]. Species found at higher abundance in feces from health rabbits included the following: *Akkermansia* sp., *Bacteroidales* sp., *Lachnospiraceae* sp., *Odoribacter* sp., *Papillibacter* sp., *Paraprevotella xylaniphila* and *Ruminococcus* sp. In contrast, animals that had a diseased digestive tract were found to have elevated levels of *Anaerotruncus* sp., *Bacillus uniformis*, *Lachnoclostridium* sp. and *Subdoligranulum* sp. This shows the complexity, and potential conflict, of data being used as biomarkers, with *Akkermansia* sp. being less abundant in the rabbits with generic digestive disorders, but more abundant in those specifically affected by ERE.

Thus far, discussions regarding the use of fecal biomarkers in rabbits have been confined to comparing healthy versus unhealthy animals, particularly in the case of animals suffering from ERE. However, fecal markers can also be associated with weaning weights. For example, 50 different operational taxonomic units (OTUs) showed significant variation in abundance based on the weaning weight [177]. Those OTUs that showed a positive association with weaning weight were often members of the Ruminococcaceae family, a family that has been shown to produce butyrate during fiber and polysaccharide fermentation [178], with butyrate acting as a source of energy for the host’s colonic epithelial cells, as well as microbes of the digestive tract [179], and also stimulating goblet cells to secrete mucins and protect the intestinal barrier [145,146,147]. Thus, screening for those organisms that have a positive association with weaning may provide a set of biomarkers to help determine if an animal is ready for weaning or not.

Other examples of using biomarkers to assess the well-being of the animal can involve applying them to look at the impact of fiber in the diet [180]. In this study, animals were fed either a high (normal) level of fiber or a low level of fiber in the diet, and then, various parameters were analyzed. Based on the use of principal component analysis, around two thirds of the variation could be accounted for by three of the variables, with the major single component being the copy number of 16S rRNA sequences detected. The sequences showed a positive correlation with short-chain fatty acid concentrations and the oxidation-reduction (redox) potential (Eh) and negatively correlated with the pH.

### 6.4. Horses

Even with the increased movement towards mechanical power, horses still play a major role in farming, agriculture and the rural environment across the globe, both as a beast of burden but also as a meat animal in some countries. Understanding the biology of their nutrition is key to their function, with several digestive disorders posing frequent problems. Consideration will be given to markers that may help with the diagnosis of conditions such as equine grass sickness, equine inflammatory bowel disease, colitis, laminitis and equine metabolic syndrome. Since the horse is a hindgut fermenter, it has been proposed that feces may provide a non-invasive and broadly reliable indicator of events in the cecum and colon. The merits and limitations of this being a valid approach have already been studied and examined (e.g., [181]). Horses are viewed as a species where dietary changes need to be introduced gradually, as there can be a dietary risk associated with it, with bacterial variation being detectable in response to dietary-induced dysbiosis [182].

Equine grass sickness (EGS) is a condition that can prove fatal and annually accounts for the death of between 1% and 2% of the horse population in the UK [183]. Although the causal agent of EGS remains unknown, it is thought that a neurotoxin produced by *Clostridium botulinum* within the digestive tract plays a role in producing the symptoms. In terms of looking for biomarkers, it was noted that there was a significant reduction in the number of bacterial species present in fecal samples collected from animals suffering from EGS versus control groups. A drop in the number of detectable bacterial species was a phenomenon that had been reported previously in another digestive condition in horses, namely colitis [184]. It is postulated that this reduction in bacterial diversity negatively affects the ability of an animal’s microbiome to display competitive exclusion, thereby potentially allowing bacterial pathogens such as *C. botulinum* to become established [183]. By comparing fecal samples from animals suffering from EGS against control animals, it was found that, proportionally, horses with EGS had a higher relative abundance of the phyla Bacteroidetes and Proteobacteria, whilst the control animals had a lower relative abundance of the phyla Firmicutes and Verrucomicrobia [183]. These observations are in keeping with the earlier work on colitis, where there was a relative increase in Bacteroidetes and a relative decrease in Firmicutes [184].

In total, 82 bacterial OTUs were identified in the work on EGS, which showed significant differences between fecal samples collected from animals suffering from EGS versus control groups, with 20 of these being viewed as possible biomarkers for EGS. Of these, almost half were from the class Clostridia (9), with the remainder being split evenly across four other classes: Gammaproteobacteria, Fusobacteria and Bacteroidia (three each), and the remaining two being members of the class Deltaproteobacteria. At the level of the OTU, four candidates were identified as being possible biomarkers for EGS (*DesulfovibrioD168*, *Enterobacteriales*, *Fusobacteriales* and *Megasphaera*). However, although these species were not found in matched controlled animals (horses maintained on the same pasture as those suffering from EGS), they were also not always found at high levels in affected animals, suggesting that either there can be more than one bacterial species individually responsible for EGS or that it arises from a combination of factors from more than one species, with more than one species able to make the relevant contributions.

The bacterium *Clostridioides difficile* is an organism that has been shown to be associated with both colitis and diarrhea in horses [185], with foals more likely to be colonized by *C. difficile*, although the bacterium can also affect older horses [186]. It has been shown to exist at a low percentage in all horses, without leading to any clinical symptoms [187]. However, in an analysis of 10 horses with diarrhea and 10 without, fecal samples from all 20 animals failed to provide material from which *C. difficile* could be isolated [186]. Instead, they reported that there was a lower bacterial diversity level in the animals that had diarrhea. In addition to the analysis of the 20 animals, larger sampling was performed on 52 horses admitted to a veterinary hospital with gastrointestinal problems. In this analysis, three horses were found to be carriers of *C. difficile*, although none of these admitted were suffering from diarrhea, but rather two presented with colic and one with proximal enteritis. Thus, this is an example of where a candidate organism has been identified for its association with diarrhea but has the potential to lead to both false positive and false negative results when being considered as a potential biomarker for diarrhea.

As with many other species, inflammatory bowel disease (IBD) can be a problem in horses and has started to attract more research interest in recent years. IBD has the potential to result in diarrhea and consequently can cause weight loss [188]. At present, the most reliable methods of confirming a diagnosis of IBD involve invasive analysis, normally at a veterinary hospital. Hence, efforts are being made to develop non-invasive alternative forms of analysis, including studying the fecal microbiome to obtain biomarkers. There was no difference between two different groups of animals with IBD relative to control horses in terms of alpha-diversity. However, at the level of individual OTUs, there were differences detected between the control group and both of the groups with IBD, although this may have been a reflection of there being differing stages of disease in at least one of the IDB groups of affected animals. However, 40 different OTUs were detected, which were differentially abundant in animals with IBD. In one IBD group, there was an increase in the numbers of members of the phylum Firmicutes relative to the control group, while in the other IBD group, there was an increase in the number of members of the phylum Actinobacteriota.

Equine Metabolic Syndrome (EMS) is a condition that is associated with insulin dysregulation, often with obesity, and can lead to an increased predisposition to develop laminitis [189]. The identification of insulin dysregulation is regarded as the definitive measure of EMS but involves collecting blood samples rather than the non-invasive methods analyzed using feces in this review. However, it has also been shown that it is possible to demonstrate significant differences in the microbial population of a group of Connemara ponies suffering from EMS relative to those that were healthy [190]. This involved a reduction in species richness in the EMS ponies, although the differences were not statistically significant. However, at the level of individual families, statistically significant differences were detected. The animals suffering from EMS showed a significant reduction in the number of nine different families, namely Anaerovoracaceae, Desulfovibrionaceae, Erysipelatoclosridiaceae, Erysipelotrichaceae, Muribaculaceae, Selenomonadaceae, SZUA-567 (members of the Firmicutes phylum), UBA3830 (members of the Pseudomonadota phylum) and UMGS1810 (members of Firmicutes phylum). Only one family showed a statistically significant increase in numbers in the EMS group, Salinicoccaceae. Differences were also detected at the genus level, with 10 genera being reduced in the EMS group and 4 genera being increased. Thus, there is potential for a set of diagnostic biomarkers in the feces, in addition to having to rely on collecting blood samples.

Possibly one of the most complex conditions associated with dietary issues in the horse is laminitis. Although it manifests as a condition in the hoof, it is generally as a consequence of dietary problems. As mentioned above, animals that suffer from EMS can have an increased risk of developing laminitis.

By feeding an animal high levels of oligofructose (around 10 g per kg of body weight), it is possible to induce laminitis. In an experiment where animals had laminitis induced there was a significant reduction in the richness of the community and the bacterial diversity in animals with laminitis relative to the control animals [191]. At the level of individual phyla, the following phyla were found to be significantly less abundant in the animals with induced laminitis versus the control group: Elusimicrobia, Fibrobacteres, Kiritimatiellaeota, Lentisphaerae, Planctomycetes, Tenericutes and Verrucomicrobia. At the level of the genus, a number of genera were found to have decreased in relative abundance, namely, *Alloprevotella*, *Akkermansia*, *Candidatus_Soleaferrea*, *Elusimicrobium*, *Fibrobacter*, *Oribactrium*, *Papillibacter* and *Phascolarctobacterium*. However, conversely, some genera increased their relative abundance in the laminitic animals: *Allisonella*, *Lactobacillus* and *Megasphaera*. These bacterial population changes are associated with increased lactic acid production, a phenomenon observed in animals that have had laminitis induced by oligofructose. In addition, a number of metabolites had their abundance altered in a manner that allowed discrimination between animals with laminitis and those not affected, based on their two principal components in a principal component analysis approach [191]. However, throughout this work, the observations dealt with samples collected from the digestive tract, rather than fecal material. Therefore, although these may yet prove useful for non-invasive biomarkers, further work would be necessary before using them for this purpose. However, while metabolites might provide an indication of the laminitic status, an investigation that compared animals that had previously been laminitic with those that had no such history found no significant differences between the two populations [192]. This suggests that even if there is a method to differentiate between animals with laminitis and those without, then detection using the method does not persist after recovery, suggesting that it may not be suitable as a method for predicting predisposition to developing the condition.

A summary of some of the biomarkers from the different species is provided in Table 1.

## 7. Biomarkers Not Involving Gut Microbes

In pigs, non-specific colitis can be observed even when it is not possible to find specific pathogens and is a condition that mainly affects growing pigs (12–40 kg, approximately 4–12 weeks post-weaning). This colitis is characterized by subacute colitis with mucosal hyperplasia, mononuclear cell infiltration, multifocal mucosal erosions, colonic lesions of an increased crypt depth and poor growth rates, mainly because of the impaired absorptive functionality and dehydration due to diarrhea [112,193]. Panah et al. [112] produced an extensive review showing that dietary factors can develop or induce colitis. These include compounds such as trypsin inhibitor, vitamins C and E, glutathione, ubiquinol, polyphenols and β-carotene, essential amino acids, dietary protein, soluble non-starch polysaccharides, resistant starch and pelleted diets, all of which, in different ways, can facilitate pathogenic infections in the large intestine and also describe possible biomarkers related to colitis-complex diarrhea. One of the most promising pair of biomarkers in pigs affected by colitis are calprotectin and lactoferrin. Calprotectin is a protein in the inflammatory response that is released by neutrophils when there is damage in the gut. Lactoferrin is an important 80 kDa Fe-binding protein present in mammalian milk and in several secreted fluids such as saliva and mucosal secretions, which can bind to pathogenic bacteria. Calprotectin has been found in the feces of adult pigs in the same range as in healthy adult humans, and since it was lower at birth in piglets, they concluded that calprotectin can be a non-invasive marker of gut inflammation in the porcine species. Barbosa et al. [194] investigated the usefulness of fecal calprotectin as a biomarker of intestinal inflammation in *Brachyspira hyodysenteriae*- and *Salmonella* Typhimurium-infected pigs. Fecal calprotectin levels increased following the development of colitis but did not significantly change due to enteritis. In the case of lactoferrin, it binds to pathogenic bacteria, thereby preventing epithelial attachment and infection.

## 8. Other Uses for Gut Biomarkers

However, biomarkers obtained from fecal samples are not only restricted to providing information regarding the health, or otherwise, of the digestive tract. It has also been shown that biomarkers obtained from feces can be used as an indicator of heat stress in a number of species, including monogastric species. Heat stress at the level of the whole body can result in damage to the intestinal structure, which historically would have been detected using histological samples. The damage to intestinal tissue can lead to dysbiosis taking place, leading to changes in the microbial community and subsequent changes in the metabolites (e.g., SCFAs) that they produce [195,196]. Moreover, effects on the digestive microbiome are linked to a range of different factors, which can extend to conditions such as irritable bowel disease, and conditions that can then impact on the gut–brain axis [196]. Thus, the consideration of fecal biomarkers has the potential to extend beyond purely the health of the digestive tract but actually has potential to allow studies of the health of numerous aspects at the whole-body level.

In commercial intensive pig-farming systems, pigs are exposed to various stress factors, which cause stress and an inflammatory response, having a negative impact on a pig’s normal physiology, welfare and overall productive performance. Multiple biomarkers have been described to detect stress and improve stress management in pig farming [197,198]. In pig-production systems, sources of stress can be categorized into environmental handling/management and social causes [199]. Studies of the microbiome–gut–brain axis performed in mice have showed that stress and the microbiome are linked [200,201].

Understanding the relationship between social stress and the gut microbiota can improve animal welfare and health. Hu et al. [195] induced intestinal inflammation in heat-stressed pigs, performed fecal microbiota transplantation from pigs to mice and explained the role of microorganisms in inflammatory bowel disease. They found that the colonic microbiome composition of heat-stressed pigs was different from that of control pigs. By day 14, *Proteobacteria*, *Lactobacillales*, *Cyanobacteria* and *Actinobacteria* and also opportunistic pathogens such as *Campylobacterales* had increased in heat-stressed pigs. The composition of the colonic microbiome in mice administered feces from heat-stressed pigs was different from those receiving control pig feces. *Bacteroides* were significantly diminished, *Akkermansia* were significantly increased and intestinal damage and goblet cell numbers were higher in mice that received feces from heat-stressed pigs. Nguyen et al. [202] used a porcine stress model based on repeated regrouping and reduced space allowance during the last 4 weeks of the finishing period, which was developed to identify stress-induced changes in the gut microbiome composition. Opportunistic pathogens such as *Treponema* and *Clostridium* were enriched in colonic and fecal samples from stressed pigs. Also, genera such as *Streptococcus*, *Parabacteroides*, *Desulfovibrio*, *Terrisporobacter*, *Marvinbryantia* and *Romboutsia* were found to be enriched in response to social stress. In addition, this provides further evidence for the microbiota–gut–brain axis, as indicated by an increase in the cortisol concentration due to social stress regulated by the hypothalamic–pituitary–adrenal axis, and a change in the microbiota composition, particularly of bacteria known to be associated with pathogenicity and mental health diseases.

## 9. Conclusions

In conclusion, we demonstrate that there is good evidence to demonstrate the potential role of fecal biomarkers in disease detection and determination across the different species investigated here. At present, this information is most widely available for pigs, in terms of the range of species investigated here, but there is also an increasing number of such markers being developed and discovered for the other species in this narrative.

## Figures and Tables

**Table 1:** Examples of biomarkers potentially associated with samples from different species.

Condition	Biomarker(s)	Reference(s)
Suckling piglets	Elevated levels of *Comamonas kerstersii*, *Comamonas aquatica* and *Roseburia inulinivorans*	[95,96]
Newly weaned piglets	Elevated levels of *Clostridium perfringens* and *Ruminococcus flavefaciens*	[95,96]
Post- weaned piglets	Elevated levels of *Prevotella copri*	[95,96]
Post- weaned piglets	Elevated levels of *Prevotella copri*	[95,96]
Neonatal piglet diarrhea	Elevated levels of *Prevotella*, *Sutterella*, *Clostridium*, *Campylobacter* and *Enterococcus*. Decreased levels of *Lactobacillus*	[42,101,102]
Dysentery in pigs	Elevated levels of *Desulfovibrio*, *Campylobacter*, *Mogibacterium*, *Fusobacterium Brachyspirales*, *Campylobacterales*, *Desulfovibrionales* and *Enterobacteriales*	[115]
Broiler dysbiosis	Elevated levels of *Clostridium perfringens* or *Escherichia coli*	[91,113,114,115]
Broiler necrotic enteritis	Elevated levels of *Lactobacillus reuteri*, *Subdoligranulum variabile*, *Mediterraneibacter* spp. and *Staphylococcus* spp. Decrease in fungal population	[127,128]
Broiler ascites syndrome	Elevated levels of Firmicutes, Proteobacteria, Actinobacteria, Firmicutes and Bacteroidetes	[140,141]
Epizootic Rabbit Enteropathy	High prevalence of *Clostridium perfringens*. Increase in Bacteroidia at the expense of Clostridia. Increased levels of γ-Proteobacteria, *Cloacibacillus porcorum* and *Akkermansia muciniphila*	[159,160,161,165]
Rabbits kept in low hygiene conditions	Decrease in family Lachnospiraceae in cecum	[170]
Equine grass sickness	Increased relative abundance of Bacteroidetes and Proteobacteria, but lower relative abundance of Firmicutes and Verrucomicrobia.	[183]
Equine colitis	Increased relative abundance of Bacteroidetes but lower relative abundance of Firmicutes	[184]
Equine inflammatory bowel disease	Increased relative abundance of Firmicutes or Actinobacteria.	[186]
Equine Metabolic Syndrome	Decrease in Anaerovoracaceae, Desulfovibrionaceae, Erysipelatoclosridiaceae, Erysipelotrichaceae, Muribaculaceae, Selenomonadaceae, SZUA-567, UBA3830 and UMGS1810 families. Increase in Salinicoccaceae family	[190]
Equine laminitis	Increased in *Allisonella*, *Lactobacillus* and *Megasphaera* genera	[191]

## Data Availability

No new data were created.
